# Effects of castration and sterilization on baseline and response levels of cortisol—A case study in male guinea pigs

**DOI:** 10.3389/fvets.2022.1093157

**Published:** 2023-01-06

**Authors:** Sylvia Kaiser, Annika Korte, Joachim Wistuba, Maximilian Baldy, Andreas Wissmann, Marko Dubičanac, S. Helene Richter, Norbert Sachser

**Affiliations:** ^1^Department of Behavioral Biology, University of Münster, Münster, Germany; ^2^Institute of Reproductive and Regenerative Biology, Centre of Reproductive Medicine and Andrology, University of Münster, Münster, Germany; ^3^Central Animal Facility, University Clinic, University of Duisburg-Essen, Essen, Germany

**Keywords:** Animal Protection Act, hormones, pets, hypothalamic-pituitary-adrenocortical axis, reproduction, stress, testosterone, welfare

## Abstract

An uncontrolled reproduction of animals in human hands should be avoided. To meet this goal, animals are widely castrated, i.e., the gonads are completely removed. Since the gonads are the most important source of sex hormones, this is a serious intervention in the entire endocrine system of an organism. Sterilization is a much less invasive procedure. Thus, it could have advantages over castration. Therefore, the overall aim of this study was to analyze the effect of castration vs. sterilization on the release of glucocorticoids, i.e., an important indicator for welfare. Taking domestic guinea pigs as a model system, we studied baseline and response cortisol values (cortisol is the main glucocorticoid in guinea pigs) in castrated, sterilized, sham-operated and intact males and baseline values in their cohoused females. Whereas baseline values of males did not differ between the groups, castrated males showed significantly higher cortisol response levels than intact, sham-operated and sterilized males. Females housed with castrated, sterilized, sham-operated or intact males did not differ in their cortisol concentrations, neither shortly after being placed with the respective male or after being co-housed for several weeks. Overall, the results support the hypothesis that castrated males exhibited a higher cortisol responsiveness during acute challenge which could point to a generalized impaired welfare of castrated males in comparison to intact, sham-operated and sterilized males. Our results provide first evidence for a potential negative impact of castration on the animals' welfare, while at the same time pointing toward sterilization representing a less invasive, promising alternative. Therefore, the results may stimulate future research on this topic to further detect potential welfare-related side effects of castration.

## 1. Introduction

Castration and sterilization are two procedures that can be used to control the reproduction of animals in human hands (1). During castration, the gonads (males: testes; females: ovaries, sometimes additionally the uterus) are removed [e.g., (2, 3)]. Since the gonads are the most important source of sex hormones such as androgens, estrogens and progestins [e.g., (3, 4)], this is a serious intervention in the entire endocrine system of an organism. After castration, only very low concentrations of these sex hormones can be secreted by other endocrine-active cells located, for example, in the adrenal cortex (3). Sterilization, on the other hand, is a less invasive intervention in the organism: here the vas deferens or the fallopian tubes are closed, cut or removed [e.g., (2)]. Sperm and egg cells can no longer be transported and thus egg cells can no longer be fertilized after this intervention and reproduction is prevented. However, the gonads are not removed and can continue to function as endocrine glands [e.g., (5)]. Therefore, this intervention has no influence on sex hormone production. Since secondary sex characteristics are mediated by sex hormones, they can develop or remain intact in sterilized males.

The primary purpose of both surgical methods is to control reproduction of animals in human hands. In pet, farm and zoo animal husbandry, however, the majority of animals are castrated, not sterilized [e.g., (1, 6–9)]. Here, it is typically argued that, in addition to preventing reproduction, castration can also prevent unwanted or excessive sexual behavior and sex hormone-induced aggression (10). Since these behaviors are controlled by sex hormones such as testosterone and estrogens (3), sterilization is often considered insufficient. However, this argument does not always hold true, because the interplay of hormones and behavior is very complex. For example, it is known that animals that are already sexually mature at the time of the procedure continue to perform courtship and sexual behaviors, or it takes a long time for these behaviors to disappear (11, 12). Furthermore, the presence or absence of sexual experience prior to castration is often critical for the decline of courtship and sexual behaviors. Regarding effects of castration on aggression, controversial results are found: some studies show that castrated animals are less aggressive than intact animals (13, 14). Other studies, however, show no differences in aggressive behavior between castrated and intact animals (15).

Another argument for castration instead of sterilization is that the risk to develop reproductive diseases (e.g., mammary gland cancer, prostate hyperplasia/infection) is reduced in castrated animals (1, 16–19). However, the risk of formation tumors outside the reproductive system, of various joint disorders (e.g., cranial cruciate ligament rupture), urinary incontinence, obesity as well as cognitive disfunctions can be increased in castrated animals (18, 20–23).

In summary, the arguments why castration should be preferred over sterilization to prevent uncontrolled reproduction are not entirely convincing. As sterilization is less invasive because the endocrine system is not disturbed, careful consideration should be given to whether this procedure should be performed instead of castration. In principle, the legislation also points in this direction. For example, according to the German Animal Protection Act, it is forbidden to perform amputations on animals without reasonable cause: “Prohibited is the complete or partial amputation of body parts or the complete or partial removal or destruction of organs or tissues of a vertebrate animal.” [§6 (1) Tierschutzgesetz 1972, last amended 2021]. Thus, according to §6 Animal Protection Act, castration of animals falls under the prohibition of amputation and may only be performed if there is a reasonable reason. Currently, medical indications and the prevention of uncontrolled reproduction count as reasonable justification in Germany. In many other Western Countries similar regulations are applied (24). However, there are also a few countries, in which the prevention of uncontrolled reproduction is not considered a sufficient reasonable reason to castrate animals. E.g., in Norway it is prohibited to castrate pet animals if the sole aim is to prevent offspring (24). Furthermore, in Sweden there is a long tradition not to castrate dogs: Salander et al. (25) described that 99% of dogs were left intact in Sweden.

Uncontrolled reproduction should often also be avoided from the animal's point of view. For example, stray dogs and cats, which are often left to their own, live under difficult conditions, especially in urban areas. This can lead to reduced welfare of the animals and therefore reproduction of these animals should be prevented. However, this goal can also be achieved by sterilization, i.e., it is not necessary to perform an ambutation. Thus, it is necessary to comparatively examine the influence of these two interventions—castration vs. sterilization—on the welfare of the animals. To assess animal welfare, several indicators are available and well-validated. Such indicators include behavioral, emotional, cognitive, and physiological parameters [e.g., (26)]. Reliable physiological indicators for animal welfare can be, for example, heart rate, body temperature, immune parameters, and the amount of glucocorticoid release [e.g., (26, 27)] with the latter being a frequently used and well-validated physiological indicator of welfare. One main function of glucocorticoids is the maintenance of homeostasis, which is ensured by the release of glucocorticoids on a continuous low baseline level (28). Baseline glucocorticoid levels depend on various factors such as nutritional state or activity level (29–31). During challenging situations, however, higher amounts of glucocorticoids are produced and secreted into the blood stream *via* activation of the hypothalamic–pituitary–adrenocortical (HPA) system, one of the main stress systems in vertebrates (32). Glucocorticoids activate various pathways that lead to activation of gluconeogenesis and redistribution of the immune system before levels return to baseline through negative feedback loops (28, 33–35). Thus, the activation of the HPA system provides the organism with energy during challenging situations and shifts it into a state of heightened reactivity that is a prerequisite for responding to environmental changes in an appropriate way. Since glucocorticoid responsiveness correlates with the maximum release of glucocorticoids after stimulation of the adrenal glands with adrenocorticotropic hormone [ACTH; (32, 36)], this can serve as a good indicator of the activity of the adrenal cortex and the secretory capacity of an individual (32). Therefore, both parameters, baseline and response values, are valid measures in stressful situations to assess animal welfare.

Although most of the studies investigating the effects of castration have been carried out on dogs and partly on cats, we used the domestic guinea pig in our study. These animals are a suitable model system for this purpose for two reasons: (1) Guinea pigs are popular pets. In the United Kingdom, for example, 0.7 million animals are kept as pets and in the USA 1.36 million animals (37). (2) The effects of stressors on glucocorticoid concentrations are known in several species [e.g., (38, 39)]. However, guinea pigs are one of the best studied species regarding the effects of stressors on baseline glucocorticoids [in the domestic guinea pig mainly cortisol; (40, 41)] and response values [e.g., (29, 36, 42–48)].

The overall aim of this study was to analyze baseline and response levels of cortisol in castrated, sterilized, sham-operated and intact male guinea pigs. Although for a broader assessment of animal welfare a wide range of indicators are desirable, baseline and response levels of cortisol are well established indicators of animal welfare and thus our approach can provide some first evidence for the different surgeries affecting the animal's welfare in different ways. Sham-operated and intact males served as controls. Since castrated males had their endocrine glands removed and thus the endocrine system was strongly affected, we predicted that castrated males would have different baseline cortisol concentrations as well as a different cortisol responsiveness than sterilized, sham-operated and intact males. In addition, the testosterone concentration in castrated males should be lower than in the other males. Besides the investigation of hormones in the males, we also investigated cortisol values of females, living together with these males. Since castration has been shown to change the odor of a male due to low testosterone levels (49), the presence of intact and sham-operated and most likely sterilized males (behaving and smelling like intact males in contrast to castrated males) is very likely to lead to a reduction in aggressive behavior between females (50). Thus, we hypothesized that females living together with castrated males have higher cortisol values.

## 2. Methods

### 2.1. Animals and housing conditions

The animals used in this experiment were bred at the Department of Behavioral Biology at the University Münster from a breeding program of multi-colored shorthaired guinea pigs (*Cavia aperea* f. *porcellus*) that were originally acquired from a breeder in 1975. All animals were born in small groups of one male, two to three females, and their pre-weaned offspring.

The used animals were weaned at about 21 days of age and transferred to either same sex groups (females; 2–12 individuals) or same-sex pairs (males) until the start of the experiment. During the experiment, each male was housed together with two females. They were housed in enclosures measuring 1 m × 1 m, with walls constructed of wood with a plastic red section at the bottom, in total 0.5 m high. The floor was covered with wood shavings (Tierwohl Super), and food (Höveler Meerschweinchenfutter 10700) and water with ascorbic acid was available *ad-libitum*. Hay was replenished daily. Each enclosure contained two shelters; one large red plastic shelter and one small red plastic or black wooden shelter. Lights were on daily from 7:00 to 19:00 and room conditions were ~22°C with about 50% humidity. At the end of the experiment, females remained at the Department of Behavioral Biology. Therefore, it could be verified whether or not the females became pregnant during the experiment. All females of groups with intact and sham-operated males became pregnant. All females housed together with castrated males did not become pregnant; females of 10 out of 14 groups with sterilized males, however, became pregnant (one sterilized male mated first 1 year later with females). Probably, a recanalization occurred in these animals. This is also known from other studies; however, to a much lower extent [e.g., (51, 52)]. One factor for a successful sterilization is the surgical procedure. Sterilization failure is observed more often when sterilization is performed without cauterization. In our study, only ligation, but no cauterization was used (see below, Section 2.4). For this reason, we had to exclude these 10 males from our analysis, since we could not know at what time recanalization occurred. Thus, we could analyze data of only 4 sterilized males. In future, it is strongly recommended to perform sterilization with cauterization and ligation.

### 2.2. Experimental procedure

The males had been castrated (*n* = 14), sterilized (14, only 4 remained sterilized) or sham-operated (*n* = 8) 30–45 days after birth, i.e., several weeks before sexual maturity, which is reached in male guinea pigs at about 70 days of age. Furthermore, intact males (*n* = 10) were also included. The castration procedure involved castration through the scrotum with immediate ligation of the blood vessels and suturing of the scrotum after the testis were removed. The sterilization procedure that was used involved excising an 8–10 mm piece of each of the two vasa deferentia after performing ligations on both ends to keep the vas closed (for details see below Section 2.4). During the experiment each one male (age about 85 days of age; range: 78–92 days of age) was introduced into a new enclosure together with two females (age of females about 75 days of age; range: 55–107 days of age; age differences between two females of one group: 0–40 days). Since each two females were housed together with one male, the sample size of females was the following: females living together with a castrated male: *n* = 28, with a sham-operated male: *n* = 16, with a sterilized male: *n* = 8, with an intact male: *n* = 20.

### 2.3. Timeline

At about 34 days of age (range: 27–41 days of age) a cortisol response test (CRT; see below) was performed on each male prior to surgery to ensure that there were no differences between the males that were later treated differently at this time. With the age of about 85 ± 7 days (around sexual maturity) each one male was introduced together with two females into a new enclosure. 5 ± 1 day before this introduction a further CRT was conducted to assess initial values. Two further CRTs were performed 8 ± 2 days and 21 days ± 2 after introduction into the groups to assess the ability to cope with this new situation in the long term. Furthermore, a blood sample of each male was taken just before as well as 2 h after introduction.

Blood samples were also taken from the females (see below for procedure): 1 day before the females were put to the male to determine baseline values, 3 h as well as 1 day after being put together with the male to determine the acute cortisol response, and about 1 week later and on the very last day to determine the long-term cortisol response.

### 2.4. Surgery

Surgeries were conducted under mixed anesthesia with ketamine and medetomidine i.m. (Ketamin^®^, Ceva Tiergesundheit GmbH, Düsseldorf, Germany, 60 mg/kg body weight; Domitor^®^, Pfizer, Berlin, Germany, 0.25 mg/kg body weight). In addition, local anesthesia was applied using a 0.5% lidocaine solution (Xylocain^®^ 1%, AstraZeneca, Wedel, Germany, in physiological saline, 0.05–0.1 ml s.c. in each side of the scrotum). Effects of medetomidine were reversed using atipamezole (Antisedan^®^, Pfizer, Berlin, Germany, 0.75–2 mg/kg body weight s.c.) after completion of surgery (at least 45 min after induction of anesthesia). Postoperative analgesia was provided by carprofen (Rimadyl^®^, Pfizer, Berlin, Germany, 4 mg/kg body weight s.c., after induction of anesthesia).

Castration: The scrotum was opened on both sides with a small incision, the testicles and epididymis were placed in front of the scrotum, ligated with absorbable suture material and removed. The scrotum was sutured in two layers (peritoneum with muscle layers and skin).

Sterilization: The abdominal cavity was opened on both sides in the groin with a small incision through the skin and abdominal wall, the vas deferens were placed in front of the abdominal cavity, and a pair of forceps was slid underneath to stretch the vas deferens open. Two surgical knots were placed between the legs of the forceps. In addition, the area between the nodes was cut out (~5–10 mm). The surgical access was then also sutured in two layers (peritoneum with muscle and skin).

Sham operations: The scrotum or abdominal cavity was opened at the same location and then closed again as described.

### 2.5. Blood sampling

In male and female guinea pigs blood samples were taken to determine cortisol concentrations. In males, furthermore testosterone concentrations were determined.

In short, guinea pigs were undisturbed for 1 h prior to the onset of the sampling procedure which always started at 9.00 a.m. The guinea pig was then removed from his/her enclosure, and a marginal ear vein was punctured to collect about 50 μl of blood within 3 min (cortisol) and further 100 μl within 6 min (testosterone). Guinea pigs show little defense behavior during the collection procedure, and no elevation of plasma cortisol occurs for about 5 min (36). Accordingly, all samples for determination of cortisol levels were collected within 3 min of entering the room to make sure that no elevation of cortisol had yet occurred. After that the guinea pig was weighed.

### 2.6. Cortisol response test

With male guinea pigs several cortisol reaction tests were conducted. During this test 3 blood samples were taken. The first sample was taken as described above at 9.00 a.m. Instead of returning the animal to its home enclosure, it was placed singly in a 1 m^2^ novel enclosure without shelter, which is a stressor for guinea pigs and triggers an increase in circulating cortisol (42) until exactly 1 h after the first blood sample (at 10.00 a.m.) was collected. This process was subsequently repeated until three blood samples were taken over 2 h at 11.00 a.m. (baseline, after 1 h, after 2 h) and then the guinea pig was returned to the home enclosure. Cortisol concentrations were determined from each blood sample; testosterone concentrations were also determined from all samples collected before start of the test.

### 2.7. Determination of hormone concentrations

The blood plasma was extracted directly after sample collection *via* centrifugation and then frozen at −20°C. Once all samples were collected at the end of the experiment, the concentrations of cortisol and testosterone were determined in duplicate using enzyme-linked immunosorbent assays (Cortisol ELISA, RE52061, IBL International GmbH, Hamburg, Germany; intra- and inter-assay CVs: 3.0 and 3.5%; antibody cross-reactivity: cortisol 100%, prednisolone 30%, 11-desoxycortisol 7%, cortisone 4.2%, prednisone 2.5%, and corticosterone 1.4%; testosterone ELISA, RE52151, IBL International; intra- and inter-assay CVs: 4.6 and 5.7%; antibody cross-reactivity: testosterone 100%, 11β-OH-testosterone 8.7%, 11α-OH-testosterone 3.2%, and dihydrotestosterone 1.9%). The technical assistant who performed the assays was blinded to the experimental design. In addition, all samples from one male were analyzed in the same assay. Furthermore, it was ensured that samples from at least three differently treated males were analyzed in the same assay.

### 2.8. Statistics

All statistics were calculated using SPSS for Windows (SPSS, Chicago, IL, USA version 28). R 4.0.0 (53) and RStudio (Posit, Boston, MA, USA, Version 2022.07.1+554) was used to create graphs. We conducted an a priori sample-size calculation using the software G^*^Power (statistics: one-way ANOVA for fixed effects). We based our calculations on the baseline and response cortisol values. Previous data showed that effects of the social environment on cortisol concentrations are large, e.g., effect sizes for large effects between *f* = 0.69–1.6 [e.g., (42, 54)]. To detect effects with *f* = 0.69 with a power of >80% a total sample size of at least 28 animals would be needed i.e., 7 per group. To detect large effects, already starting at *f* = 0.4–0.5 with a power of >80%, we decided to use 14 animals per group, resulting in a total sample size of 56 animals, i.e., 14 animals per group. The calculated sample size sometimes deviates considerably from the achieved sample size. In the case of sterilized animals, this is due to the surgery (see Section 2.1). Furthermore, at some time points too few males were born for four treatment groups. Thus, the sample size was the following: sterilized males: *n* = 4 (see Section 2.1 for reason for the small sample size), castrated males: *n* = 14; sham-operated males: *n* = 8; intact males: *n* = 10. Because the sample size was very small and measures violated assumptions for parametric tests and transformations failed to correct this, non-parametric statistics were used. Kruskal-Wallis tests were used to determine whether there were any treatment effects in testosterone, baseline and response values of cortisol between animals of the four groups. In case of significance, pairwise comparisons between castrated, sterilized, intact and sham-operated males were subsequently performed using Mann–Whitney *U*-tests (Holm-Bonferroni corrected). In general, two-tailed tests were used and significance was set at *p* < 0.05. Central tendency and variability for endocrine measures were expressed as medians and maximum and minimum values.

## 3. Results

### 3.1. Testosterone concentrations of males

**Figure 1:**
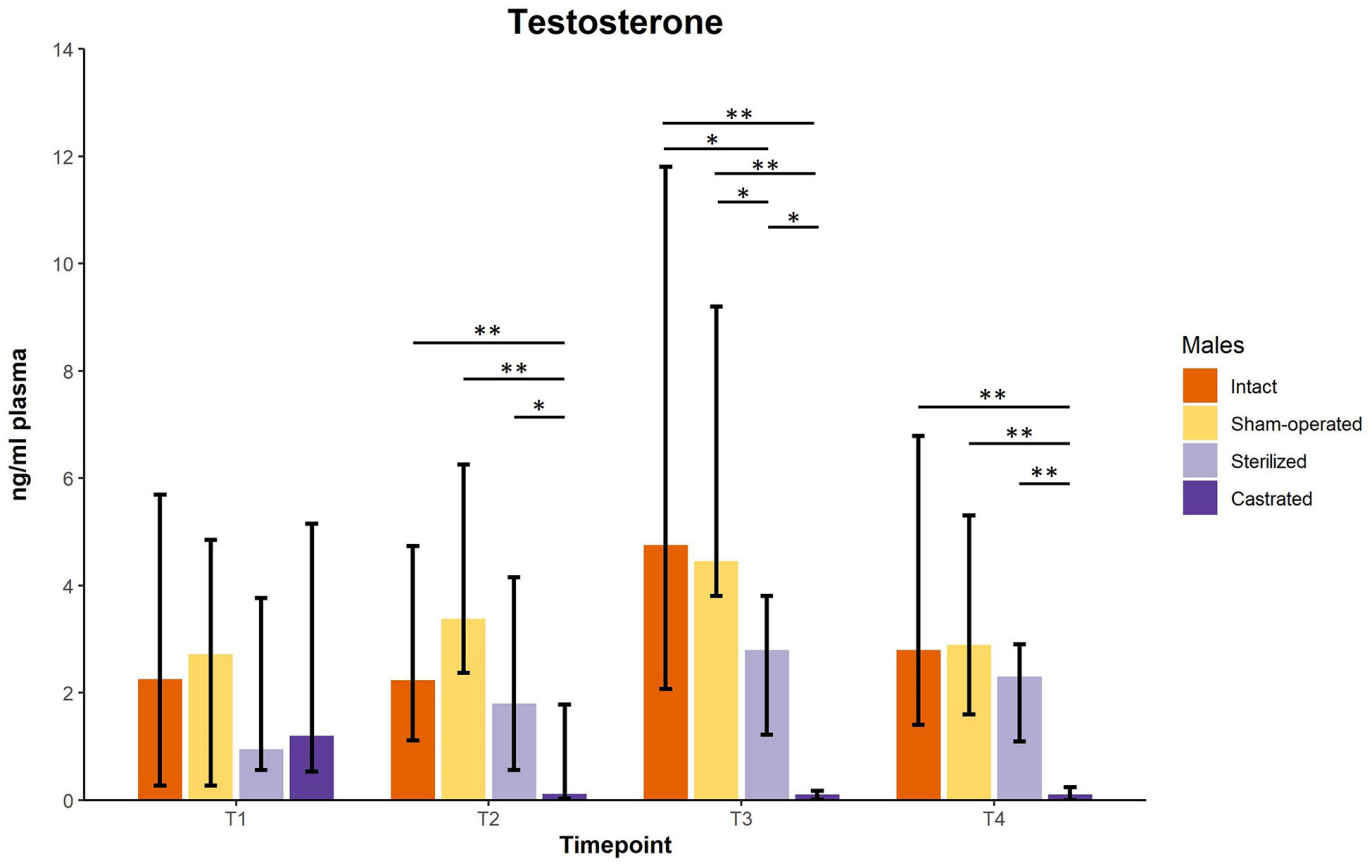
Testosterone concentrations in ng/ml plasma of intact, sham-operated, sterilized and castrated males. Data are presented as medians, minimum and maximum values. T1, testosterone concentrations before surgery; T2, testosterone concentrations about 5 days before start of experiment (1 male and 2 females were put together in a new enclosure); T3, testosterone concentrations 1 week after introduction into the new enclosure; T4, testosterone concentrations 2 weeks after introduction into the new enclosure. Statistics: Kruskal-Wallis tests, *post-hoc* Holm-Bonferroni corrected Mann-Whitney *U*-tests. *N*_intact_ = 10, *n*_sham−operated_ = 8, *n*_castrated_ = 14, *n*_sterilized_ = 4. *p < 0.05, **p < 0.01 (after Holm-Bonferroni correction).

Testosterone concentrations did not differ between males before surgeries at the age of about 34 days. However, there was a significant difference of plasma testosterone concentrations between intact (*n* = 10), sham-operated (*n* = 8), castrated (*n* = 14) and sterilized (*n* = 4) males after surgery at each sample point (see Figure 1; for statistical details see Table 1). *Post-hoc* tests revealed that castrated males showed lower testosterone concentrations than intact, sham-operated and sterilized males (Figure 1; for statistical details see Table 1).

**Table 1:** Statistical parameters of testosterone concentrations of intact (*n* = 10), sham-operated (*n* = 8), castrated (*n* = 14), and sterilized (*n* = 4) males.

	**Kruskal-Wallis**	**Mann Whitney** ***U*****-test** **U** **P**					
	**Hp**	**Intact-sham**	**Intact-castr**.	**Intact-steril**.	**Sham-castr**.	**Sham-steril**.	**Castr.-steril**.
T1	**2.607**						
	**0.456**						
T2	**25.735**	18	**3**	14	**0**	5	**2**
	**<0.001**	n.s.^*^	**0.01** ^*^	0.454	**0.01** ^*^	n.s.^*^	**0.05** ^*^
T3	**27.849**	36.5	**0**	**4.5**	**0**	**0.5**	**0**
	**<0.001**	n.s.	**0.01** ^*^	**0.05** ^*^	**0.01** ^*^	**0.05** ^*^	**0.05** ^*^
T4	**25.804**	33.5	**0**	13	**0**	8.5	**0**
	**<0.001**	n.s.	**0.01** ^*^	n.s.	**0.01** ^*^	n.s.	**0.01** ^*^

T1, testosterone before surgery; T2, testosterone about 5 days before start of experiment (1 male and 2 females were put together in a new enclosure); T3, testosterone 1 week after introduction into the new enclosure; T4, testosterone 2 weeks after introduction into the new enclosure.

* Adjusted p after Bonferroni-Holm correction; n.s., non-significant. Significant differences were highlighted in bold.

### 3.2. Cortisol baseline and responsiveness of males

**Figure 2:**
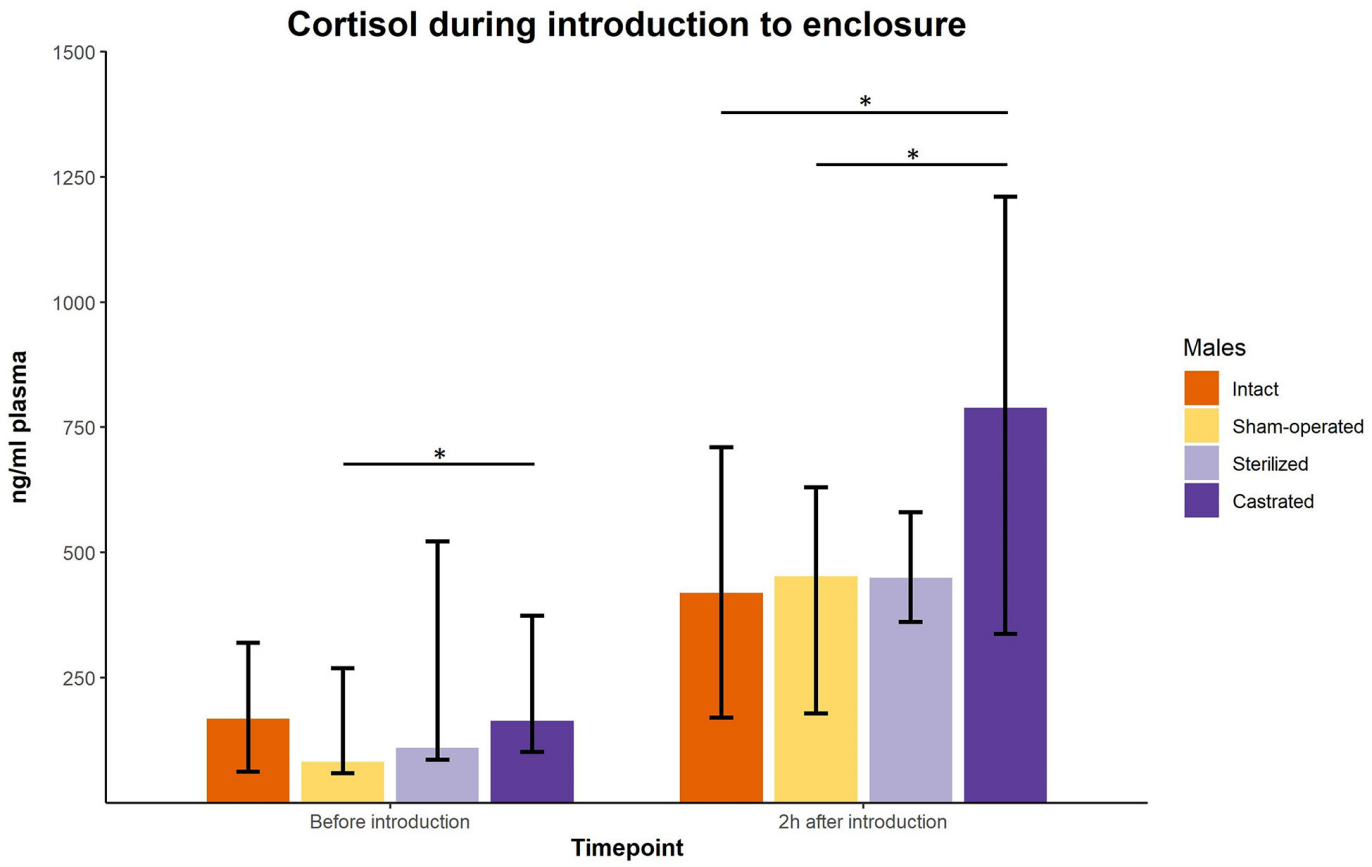
Cortisol concentrations in ng/ml plasma of intact, sham-operated, sterilized and castrated males before and shortly after introduction (1 male and 2 females were put together in a new enclosure). Data are presented as medians, minimum and maximum values. Before introduction: blood sample taken just before start of experiment; 2 h after introduction: blood sample taken 2 h after introduction in the new enclosure. Statistics: Kruskal-Wallis tests, *post-hoc* Holm-Bonferroni corrected Mann-Whitney *U*-Tests. *N*_intact_ = 10, *n*_sham−operated_ = 8, *n*_castrated_ = 14, *n*_sterilized_ = 4. *p < 0.05 (after Holm-Bonferroni correction).

Cortisol concentrations were significantly different immediately before the males were transferred to the females in a new enclosure and 2 h later (intact males: *n* = 10; sham-operated males: *n* = 8, castrated males: *n* = 14, sterilized males: *n* = 4). *Post-hoc* tests showed that the values shortly before introduction into the groups were in castrated males higher than in sham-operated and castrated males (Figure 2; for statistical details see Table 2). Two hours after introduction, cortisol concentrations were significantly higher in castrated males than in intact, sham-operated and sterilized males (Figure 2; for statistical details see Table 2).

**Table 2:** Statistical parameters of cortisol concentrations of intact (*n* = 10), sham-operated (*n* = 8), castrated (*n* = 14), and sterilized (*n* = 4) males.

	**Kruskal-Wallis**	**Mann Whitney** ***U*****-test** **U** **P**					
	**Hp**	**Intact-sham**	**Intact-castr**.	**Intact-steril**.	**Sham-castr**.	**Sham-steril**.	**Castr.-steril**.
CRT1 base	3.993						
	0.262						
CRT1 1 h	1.190						
	0.755						
CRT1 2 h	3.892						
	0.273						
CRT2 base	**9.819**	28	36	17	**15**	9	13.5
	**0.020**	n.s.	n.s.^*^	n.s.	**0.05** ^*^	n.s.	n.s.
CRT2 1 h	**20.042**	31.5	**17**	15	**2**	13	**0**
	**<0.001**	n.s.	**0.01** ^*^	n.s.	**0.01** ^*^	n.s.	**0.01** ^*^
CRT2 2 h	**20.108**	31	**22**	11	**0**	10	**0**
	**<0.001**	n.s.	**0.01** ^*^	n.s.	**0.01** ^*^	n.s.	**0.01** ^*^
CRT3 base	6.641						
	0.084						
CRT3 1 h	**20.257**	14	**14**	20	**3**	6	10
	**<0.001**	n.s.^*^	**0.01** ^*^	n.s.	**0.01** ^*^	n.s.	n.s.^*^
CRT3 2 h	**20.433**	28	**5**	18	**0**	15	12
	**<0.001**	n.s.	**0.01** ^*^	n.s.	**0.01** ^*^	n.s.	n.s.
CRT4 base	6.637						
	0.084						
CRT4 1 h	**23.257**	23	**12**	10	**0**	10	**0**
	**<0.001**	n.s.	**0.01** ^*^	n.s.	**0.01** ^*^	n.s.	**0.01** ^*^
CRT4 2 h	**22.485**	32	**10**	11	**0**	12	**1**
	**<0.001**	n.s.	**0.01** ^*^	n.s.	**0.01** ^*^	n.s.	**0.01** ^*^
Before introduction	**8.229**	19	64.5	16	**15**	7	17
	**0.042**	n.s.*	n.s.	n.s.	**0.05** ^*^	n.s.	n.s.
2 h after introduction	**12.247**	35	**20**	16	**18**	15	9
	**0.007**	n.s.	**0.05** ^*^	n.s.	**0.05** ^*^	n.s.	n.s.^*^

CRT 1, cortisol response test before surgery; CRT2, cortisol response test about 4 days before start of experiment (1 male and 2 females were put together); CRT3, cortisol response test 1 week after start of experiment; CRT4, cortisol response test 2 weeks after start of experiment; before introduction, blood sample taken just before start of experiment; 2 h after introduction, blood sample taken 2 h after introduction in the new enclosure; base, baseline values; 1 h, reaction value after 1 h; 2 h, reaction value after 2 h.

* Adjusted p after Bonferroni-Holm correction; n.s., non-significant. Significant differences were highlighted in bold.

**Figure 3:**
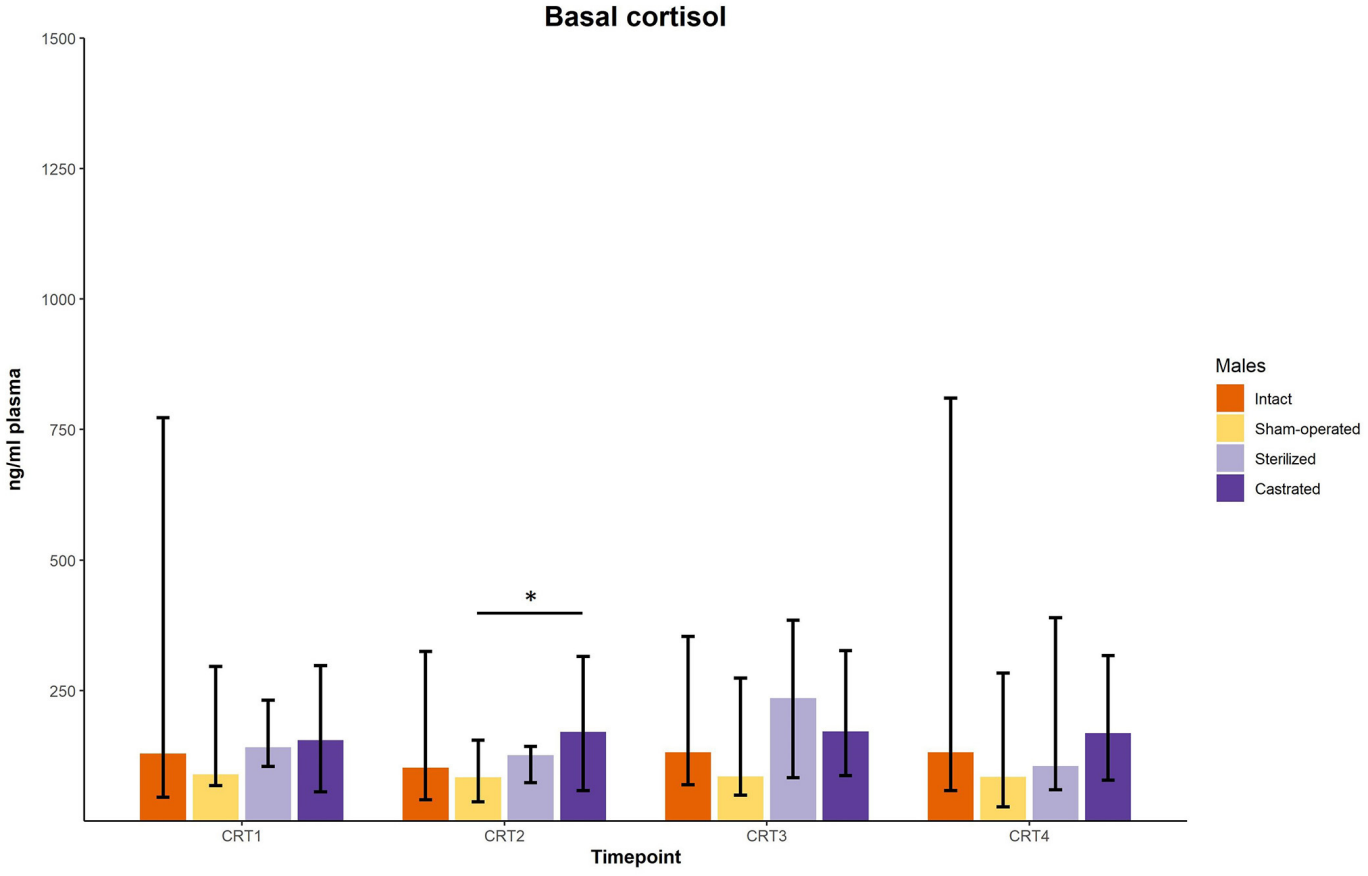
Baseline cortisol concentrations in ng/ml plasma of intact, sham-operated, sterilized and castrated males during cortisol response tests. Data are presented as medians, minimum, and maximum values. CRT1, cortisol response test before surgery; CRT2, cortisol response test about 4 days before start of experiment (1 male and 2 females were put together); CRT3, cortisol response test 1 week after start of experiment; CRT4, cortisol response test 2 weeks after start of experiment. Statistics: Kruskal-Wallis tests, *post-hoc* Holm-Bonferroni corrected Mann-Whitney *U*-Tests. *N*_intact_ = 10, *n*_sham−operated_ = 8, *n*_castrated_ = 14, *n*_sterilized_ = 4. *p < 0.05 (after Holm-Bonferroni correction).

**Figure 4:**
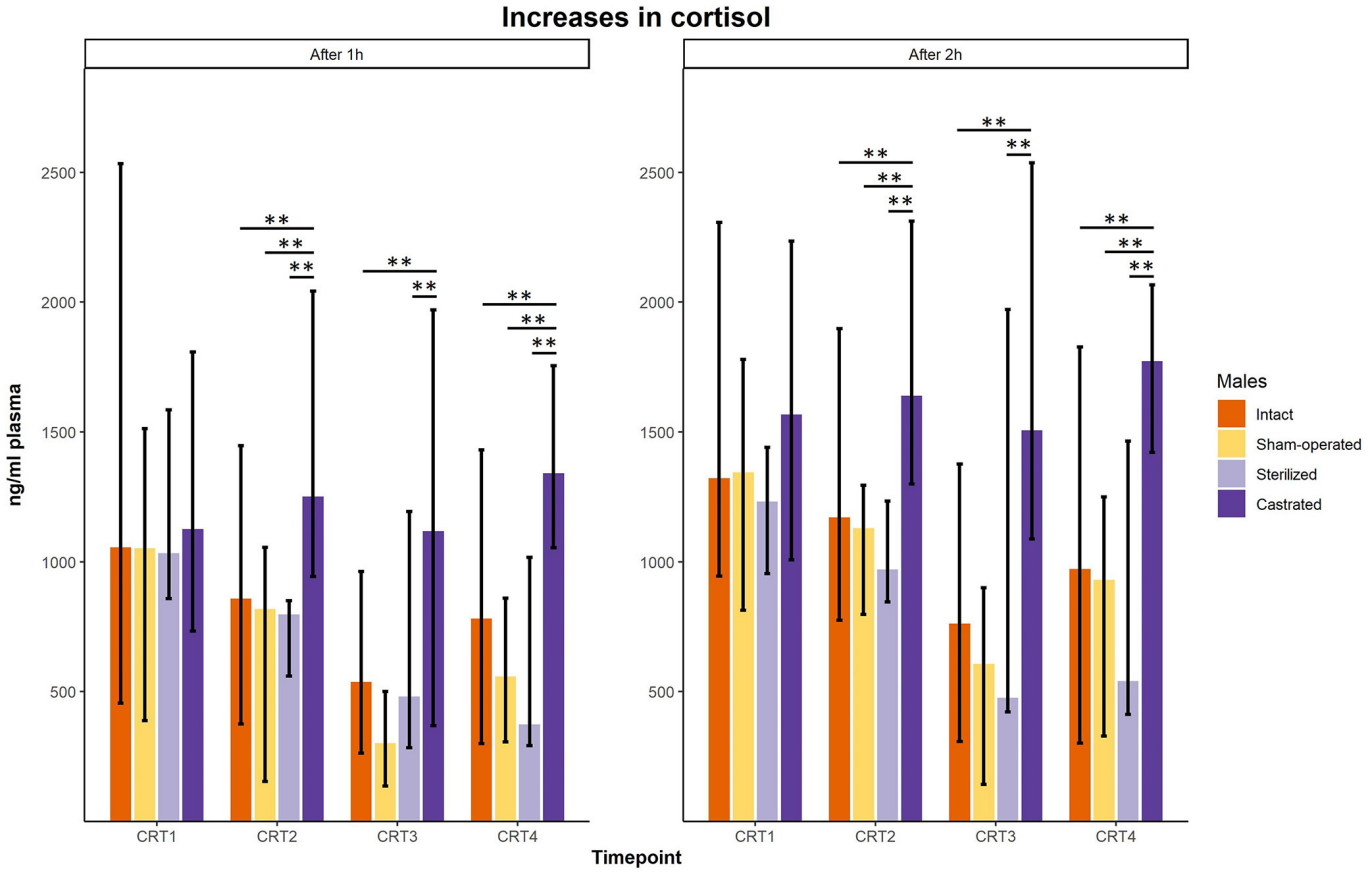
Response values of cortisol in ng/ml plasma of intact, sham-operated, sterilized and castrated males after 1 h (left panel) and after 2 h (right panel) during cortisol response tests. Data are presented as medians, minimum and maximum values. CRT1, cortisol response test before surgery; CRT2, cortisol response test about 4 days before start of experiment (1 male and 2 females were put together); CRT3, cortisol response test 1 week after start of experiment; CRT4, cortisol response test 2 weeks after start of experiment. Statistics: Kruskal-Wallis tests, *post-hoc* Holm-Bonferroni corrected Mann-Whitney *U*-Tests. *N*_intact_ = 10, *n*_sham−operated_ = 8, *n*_castrated_ = 14, *n*_sterilized_ = 4. **p < 0.01 (after Holm-Bonferroni correction).

In the cortisol response test, baseline as well as response values of cortisol was determined. Before surgeries, no differences in cortisol baseline and response values were found (Figures 3, 4; for statistical details see Table 2). One week before the start of the experiment (i.e., surgeries were conducted, but males were not yet introduced to the females), differences in baseline values were found: *post-hoc* tests revealed higher values in castrated than in sham-operated males (Figure 3; for statistical details see Table 2). Baseline levels after surgery did not differ (Figure 3; for statistical details see Table 2). Regarding response values, 1 week before as well as 2 and 3 weeks after the start of the experiment significant differences could be determined. *Post-hoc* tests demonstrated that castrated males showed significantly higher *C* response values than intact, sham-operated and on two time points also than sterilized males (Figure 4; for statistical details see Table 2).

### 3.3. Cortisol concentrations of females

In females only cortisol baseline values were determined, i.e., blood samples were taken 1 day before as well as shortly, 1 and 3 weeks after housing them together with a castrated (*n* = 28), sham-operated (*n* = 16), sterilized (*n* = 8) or an intact male (*n* = 20). Only one minor difference could be found. Cortisol concentrations were significantly different before start of the experiment (Table 3). *Post-hoc* tests showed that females that were later housed with an intact male had significantly higher cortisol concentrations than females that were later housed with a sham-operated male (Mann-Whitney *U*-Test, two-tailed, *U* = 66.5, *p* < 0.05. No differences were observed at all time points after the females. Irrespective of whether they were housed with an intact, sham operated, sterilized or castrated or male.

**Table 3:** Median, range and statistical parameters of cortisol concentrations of females living together with intact (*n* = 20), sham-operated (*n* = 16), castrated (*n* = 28), and sterilized (*n* = 8) females.

	**Intact**	**Sham**	**Castr**.	**Steril**.	**Kruskal-Wallis**
	**Median (range) in ng/ml**				**H** **p**
C0	858	546	661	509	**8.606**
	(216–1,450)	(359–785)	(147–1,644)	(158–1,533)	**0.035**
C1	985	676	738	545	6.491
	(307–1,297)	(424–1,560)	(279–2,374)	(386–1,753)	0.090
C2	719	616	736	536	2.862
	(197–1,298)	(307–790)	(188–1,223)	(277–1,303)	0.413
C3	705	604	597	639	2.190
	(234–1,488)	(230–1,300)	(282–1,400)	(265–963)	0.534
C4	775	709	668	797	0.163
	(311–1,502)	(338–2,277)	(350–1,978)	(560–850)	0.983

C0, cortisol values 1 day before introduction of the females to the male; C1, cortisol values 3 h after introduction to the male; C2, cortisol values on the day after the introduction to the male; C3, cortisol values about a week later; C4, cortisol values on the last day of experiment. Significant differences were highlighted in bold.

## 4. Discussion

In this study, we investigated whether castration and sterilization have different effects on baseline cortisol concentrations as well as cortisol response values of male guinea pigs and their cohoused females. Whereas, baseline values of males did not differ, castrated males showed significantly higher cortisol reaction levels than intact, sham-operated and sterilization males. Overall, the results support the hypothesis that castrated males show a higher stress responsiveness in acute stress situations. Furthermore, cortisol concentrations of the females did not differ, regardless of the type of surgery their cagemate has experienced.

### 4.1. Cortisol baseline and responsiveness as welfare indicator

As described in the introduction, baseline and response glucocorticoid values, are valid measures in stressful situations to assess animal welfare. In our study, cortisol baseline values did not differ between castrated males and intact, sham-operated and sterilized males (with one exception: before start of the experiment castrated males showed higher baseline values than sham-operated males). Thus, in non-challenging situations all males do not show any endocrinological signs of impaired welfare. Interestingly, however, castrated males showed higher cortisol response values after exposure to an acute stressor (novel environment, separation from their group), i.e., castrated males were more reactive in acute challenging situations than intact, sham-operated and sterilized males. This points to a less effective way to cope with stress situations in castrated than in non-castrated male guinea pigs. This could be a hint to a generalized impaired welfare in castrated males, at least in unpredictable and challenging situations. A similar finding could be shown in differently housed guinea pigs: Males housed singly showed a higher activation of the HPA system after an acute stressor than males housed together with a female. This indicates a less effective coping ability with stressful situations in guinea pigs kept alone compared to guinea pigs living in social groups (36).

Interestingly, no differences in cortisol concentrations were found in females living together with castrated, sterilized, sham-operated or intact males. This is contrary to our prediction and also to other species as for example mice. Garratt et al. (55) could show that female mice paired with castrated males had higher corticosterone concentrations (the main glucocorticoid in mice) than those females cohoused with sterilized males, thus indicating a higher stress response. These different results can possibly be explained by different forms of social organization, a different importance of males for females as well as a different importance of odors for communication.

### 4.2. Mediating effects of testosterone

Since males were castrated before sexual maturity in our study, testosterone levels were very low during adolescence. Interestingly, some studies could demonstrate that testosterone can be involved in organizing the HPA axis. In domestic guinea pigs, it was reported that the level of testosterone experienced during adolescence can shape cortisol responsiveness: if high testosterone concentrations are present during adolescence, low cortisol concentrations are released later in life during an acute stress situation. If low testosterone concentrations are present during adolescence, high cortisol concentrations are released later in life (56, 57). Thus, an inhibiting effect of testosterone on the cortisol response occurs during adolescence. It is very likely that this mechanism exists in a similar manner not only for domestic guinea pigs, but for other group-living animals (44, 58). Thus, in our study castrated males could show higher cortisol responsiveness during the cortisol response test in comparison to intact, sham-operated and sterilized males due to the mediating effects of testosterone on the HPA axis. The artificial alteration of the testosterone concentration through castration thus has far-reaching consequences for the stress reactivity of the animals. As this could be an indication of a lower coping capacity in acute stress situations, this could have implications for animal welfare.

Overall, our results point to a possibly impaired welfare in castrated animals. However, in our study we decided to focus on baseline and response levels of cortisol, which are well established indicators of animal welfare. For a broader, in depth assessment of animal welfare, other parameters must also be investigated in future studies. Besides cortisol, these could be other physiological parameters (e.g., heart rate or immunological measurements), but also ethological indicators (e.g., the extent of aggressive or stereotypic behavior) and the assessment of affective states [e.g., fear or pleasure; (59)]. Our study is limited due to the small sample size and the focus on cortisol baseline and response values. Nevertheless, the results strongly encourage further investigations on whether sterilization is not often an alternative to castration in terms of animal welfare when choosing a method to prevent uncontrolled reproduction.

## Data availability statement

The raw data supporting the conclusions of this article will be made available by the authors, without undue reservation.

## Ethics statement

The animal study was reviewed and approved by the Landesamt für Natur, Umwelt und Verbraucherschutz Nordrhein-Westfalen LANUV NRW, reference number: 84-02.04.2017.A199.

## Author contributions

SHR, NS, and SK conceived the study. AK, NS, SHR, and SK designed the experiments. AK, JW, AW, and MD conducted the surgeries. AK and MB performed the experiments, while SK supervised the project. SK analyzed the data and wrote the initial draft of the manuscript. MB created the figures. All authors revised it critically for important intellectual content. All authors contributed to the article and approved the submitted version.
